# A prospective ecological momentary assessment study of an ayahuasca retreat: exploring the salutary impact of acute psychedelic experiences on subacute affect and mindfulness skills in daily life

**DOI:** 10.1007/s00213-024-06704-8

**Published:** 2025-01-18

**Authors:** Sharon R. Sznitman, Yoel A. Behar, Sheila Daniela Dicker-Oren, Tamar Shochat, David Meiri, Nader Butto, David Roe, Amit Bernstein

**Affiliations:** 1 School of Public Health, University of Haifa, 199 Aba Khoushy Ave., Mount Carmel, P.O. Box: 3338. ZIP: 3103301, Haifa, Israel; 2 Departments Psychology and Philosophy, University of Haifa, Haifa, Israel; 3 Department of Community Mental Health, University of Haifa, Haifa, Israel; 4 Cheryl Spencer Department of Nursing, Faculty of Social Welfare and Health Sciences, University of Haifa, Haifa, Israel; 5 Faculty of Biology, Technion-Israel Institute of Technology, Haifa, Israel; 6 College of Integrative Medicine, Herzliya, Israel; 7 Observing Minds Lab, Department of Psychology, School of Psychological Sciences, University of Haifa, Haifa, Israel

**Keywords:** Ayahuasca, Psychedelics, Mindfulness, Positive and negative affect, Ecological momentary assessment, Adverse effects

## Abstract

**Rationale:**

To examine the acute effects of ayahuasca use and their relationship to sub-acute changes in affect and mindfulness in a non-clinical sample, addressing the need for a better understanding of ayahuasca’s immediate and short-term impacts as interest in its use grows.

**Objectives:**

Using prospective ecological assessment, this study investigates how ayahuasca used at a 4-day retreat affects positive/negative affect and mindfulness skills in daily living compared to pre-retreat. Additionally, we explore acute psychedelic experiences during the ayahuasca retreat, assessed retrospectively 1–2 days post-retreat, as potential mechanisms for theorized effects in daily living post-retreat.

**Methods:**

Thirty-six participants reported positive/negative affect and mindfulness skills three times daily for 5 days before and after the retreat. Baseline assessments included lifetime psychedelic experience, and post-retreat assessments covered acute ayahuasca experiences. Mixed-effect linear models were used to analyze the data.

**Results:**

Post-retreat, we observed reduced negative affect, increased positive affect, and enhanced mindfulness skills in daily living. Ayahuasca-induced acute experiences, such as time/space transcendence, emotional breakthrough and challenging experiences predicted greater subacute positive affect. Notably, none of these experiences were linked to subacute improvements in negative affect or mindfulness. No participants showed clinically significant adverse responses post-retreat, and only 5.5% exhibited some degree of potentially clinically significant deterioration in affect.

**Conclusions:**

Ayahuasca use may lead to improvement in mood and mindfulness skills, and key acute psychedelic experiences induced by ayahuasca may be important to some of these salutary effects, positive affect in particular.

**Supplementary Information:**

The online version contains supplementary material available at 10.1007/s00213-024-06704-8.

## Introduction

Ayahuasca, a psychoactive brew traditionally used by Indigenous peoples in South America for medicinal and spiritual reasons, contains DMT and beta-carboline alkaloids (Rivier and Lindgrens 1972; Majić et al. 2015; Domínguez-Clavé et al. 2016). The popularity of contemporary psychedelic movements (Pollan 2018; Johnson et al. 2019; Yaden et al. 2021) has grown globally in part due to ayahuasca tourism, expansion of ayahuasca churches, and the rise of neo-shamanic ceremonies in Western countries (Kavenská and Simonová 2015; Fotiou 2020). Ayahuasca triggers an altered state of consciousness characterized by introspective effects and vivid, dream-like visions that encompass personal and emotional memories, as well as transpersonal experiences (Bouso and Riba 2011). The onset of acute effects induced by ayahuasca occurs 45–60 min after administration, peaks in intensity between 90 and 120 min, and returns to baseline after 4–6 h (Riba et al. 2003; Dos Santos et al. 2012).

Observational studies of naturalistic ayahuasca use have shown that subacute effects secondary to ayahuasca administration, which endure or manifest following the initial acute effects, may occur even after single-time use (Uthaug et al. 2018). These subacute effects have been labeled as “psychedelic afterglow” (Majić et al. 2015) and a systematic review has estimated that they last between 1 day to 2 weeks following ayahuasca use whereas long term effects are considered to occur longer than this period (Dos Santos et al. 2016). There is growing interest in the subacute effects, in particular, as they are important for evaluating possible therapeutic applications of ayahuasca and related psychedelics.

Empirically, ayahuasca research is relatively modest in scope and rigor relative to certain other psychedelics (e.g. psilocybin) (Reiff et al. 2020; Aday et al. 2021; Golden et al. 2022; Bender and Hellerstein 2022). Within the ayahuasca literature, research has found that use of the substance is associated with various positive subacute effects, including more positive affect and less negative affect (Domínguez-Clavé et al. 2016; Dos Santos et al. 2016; Perkins et al. 2022). Research has also found that ayahuasca use, in experimental clinical and naturalistic use settings, is related to enhanced subacute mindfulness skills (e.g. reduced judgmental processing of experiences and reactivity, greater decentering) (Thomas et al. 2013; Soler et al. 2016, 2018; Domínguez-Clavé et al. 2016; Dos Santos et al. 2016).

Importantly, while significant research has been conducted on the mechanisms underlying subacute effects of ayahuasca use, there is still much to be understood. Several recent studies have made important contributions to this field. For example, Agin-Liebes et al. (2022) examined how acute factors induced by ayahuasca use, such as mystical experiences, ego dissolution, and psychological processes, moderated changes in psychological flexibility, positive affect, and negative affect over a 3-month period. Weiss et al. (2021, 2023b) investigated how a variety of acute peak and transcendent use-related ayahuasca experiences may relate to changes in personality traits and the reexperiencing of adverse life events.

Other studies have focused on only one or a few acute experiences. A randomized placebo-controlled trial in a small sample of clinically depressed individuals (*n* = 14 ayahuasca, *n* = 15 placebo) documented that ayahuasca administration led to acute ‘mystical experiences’ and subsequently to subacute anti-depressive effects (Palhano-Fontes et al. 2018). An additional cross-sectional study in a larger sample of ayahuasca users from 50 countries (*n* = 11,912), focused on a sub-sample with depression or anxiety (*n* = 2,011) and found that acute ‘mystical experiences’ were related to lower levels of subacute anxiety and depression symptoms (Sarris et al. 2021).

Similar to acute ‘mystical experiences’, emotional breakthroughs (Roseman et al. 2019) induced by classical psychedelics have also been associated with subacute outcomes. Initial studies have documented that acute psychedelic-induced emotional breakthrough may be associated with improved subacute wellbeing and reduced depression in clinical samples tested in experimental research settings as well as in non-clinical samples using psychedelics in naturalistic settings (Roseman et al. 2019; Murphy et al. 2022; Nygart et al. 2022). To the best of our knowledge, there is no published research examining acute experiences of emotional breakthroughs experiences induced specifically by ayahuasca and how these acute experiences may be tied to its documented salutary subacute effects on affect and mindfulness skills. Yet, in related work, acute ego dissolution or self-transcendence effects of ayahuasca (Nour et al. 2016) have been tied to salutary subacute effects on depression and mental health in small (*n* = 20) to modest (*n* = 50) clinical and non-clinical samples using observational survey design methods (Uthaug et al. 2018).

In addition to the above mentioned acute effects, psychedelic use has also been associated with acute challenging experiences, including affective (fear/grief/depressed mood), physiological (physical distress such as increased heart rate, nausea), and cognitive (feelings of isolation, paranoia, feelings of insanity, and the subjective experience of death) effects (Barrett et al. 2016). Observational research has found that such difficult acute experiences are commonly interpreted by participants as beneficial to their mental well-being (Carbonaro et al. 2016; Johnstad 2021; Gashi et al. 2021; Bouso et al. 2022; Lake and Lucas 2023). Weiss et al. (2021) found that higher levels of intense negative experiences during ayahuasca ceremonies, were associated with greater increases in Extraversion both immediately after the ceremony and at follow-up, as well as more substantial decreases in Neuroticism at follow-up. Yet, a randomized 6-week trial found no significant evidence that challenging experiences mediated the effects of psychedelics on depression (Weiss et al. 2024). Additionally, challenging experiences can sometimes contribute to post-acute distress, functional impairment, and medical attention seeking (e.g., Larsen 2016; Durante et al. 2020; Barber et al. 2022; Bouso et al. 2022; Bremler et al. 2023). For instance, among individuals with lifetime use of a classic psychedelic, 8.9% reported experiencing functional impairment for longer than one day, and 2.6% reported seeking medical or psychological assistance following a challenging psychedelic experience (Simonsson et al. 2023). There are also reports of the emergence of psychiatric diagnoses, suicidality, and harm to self and others during and after challenging psychedelic experiences (Carbonaro et al. 2016; Zeifman et al. 2021; Bremler et al. 2023).

### Gaps in the current literature

Despite these important advancements, there remains a need for research examining the immediate post-retreat period and the relationship between acute experiences and affect and mindfulness skills in everyday life. While several prospective studies have examined the subacute effects of ayahuasca (e.g., de Lima Osório et al. 2015; Sanches et al. 2016; Uthaug et al. 2019, 2021; Jiménez-Garrido et al. 2020; van Oorsouw et al. 2021, 2022), many have relied primarily on retrospective self-reports administered at a limited number of time points. These seminal studies have provided valuable insights, but are constrained by the limitations of retrospective reporting over a limited number of observations over time. Retrospective reporting may be particularly psychometrically problematic when assessing dynamic, variable and context-sensitive psychological states and processes such as affect and mindfulness. Affect can fluctuate rapidly, and retrospective reports may not accurately capture these variations, leading to potential recall bias (Shiffman et al. 2008; Solhan et al. 2009; Trull et al. 2015; Colombo et al. 2020). Similarly, mindfulness is a state of present-moment awareness, which can be challenging to assess accurately through retrospective measures (Bishop et al. 2004; Sauer et al. 2013; Moore et al. 2016; Shoham et al. 2017; Enkema et al. 2020; Hadash et al. 2023). The act of retrospectively recalling one’s emotional states or level of mindfulness may be influenced by current mood or recent experiences, potentially distorting the accuracy of the reports (Schwarz and Oyserman 2001; Bylsma and Rottenberg 2011).

Beyond the methodological challenges of retrospective reporting, another critical gap in the literature concerns the role of differences in psychedelic experiences. Research has found that past experiences and knowledge related to psychedelic use have important effects on the outcome of use (Aday et al. 2021), but this is rarely adequately controlled or tested for in studies examining the effects of ayahuasca (Muthukumaraswamy et al. 2021).

### The current study

The current study builds upon this existing work by employing ecological momentary assessment (EMA) to capture more frequent, real-time data on participants’ experiences in their daily lives. More specifically, we examined subacute positive and negative affect and mindfulness skills in daily living, before and after a 4-day ayahuasca retreat. EMA involves the collection of repeated, real-time data on processes and outcomes of interest, often through mobile apps or electronic diaries, in participants’ daily life. This method enables collection of repeated real-time measures of affect and mindfulness skills before and after the ayahuasca retreat, significantly reducing the recall bias that has limited previous retrospective studies (Hektner et al. 2007; Shiffman et al. 2008; Carlson et al. 2016). While previous studies have collected prospective data and sometimes ask about feelings/experiences in the last 24 h (close to real time, Soler et al. 2016, 2018; Murphy-Beiner and Soar 2020; Uthaug et al. 2021), our more frequent assessments offer several advantages. EMA enhances ecological validity by capturing experiences in participants’ natural environments, providing a more accurate representation of ayahuasca’s effects on studied outcomes, over time and across contexts of daily life. Moreover, the reported mixed models account for nested data structures, controlling for contextual factors influencing affect and mindfulness. More frequent data points provide a more robust measurement of overall trends, as naturally occurring fluctuations in affect and mindfulness are captured and accounted for, rather than potentially biasing results as they might in less frequent, single-timepoint assessments. While EMA approaches are still relatively novel in psychedelic research, a recent study by Weiss et al. (2023a) utilized EMA to examine changes in PTSD symptoms following ayahuasca use among military veterans. Our study builds and complements this work by applying EMA to a non-clinical sample and focusing on affect and mindfulness outcomes in daily life after participation in ayahuasca ceremonies. EMA designs often yield better model fit and power than designs with fewer assessments (Bolger and Laurenceau 2013). Our mixed models estimate fixed and random effects, capturing group trends and individual variability for a nuanced understanding of ayahuasca’s effects.

Moreover, we examine whether and which acute psychedelic experiences (mystical and challenging experiences, ego dissolution and emotional breakthrough) during ayahuasca use were associated with greater subacute salutary effects in daily living following the retreat. Our study includes a comprehensive battery of measures of acute psychedelic experiences. The breadth and depth of acute factors examined surpasses some of the previous studies on ayahuasca (e.g., Palhano-Fontes et al. 2018; Uthaug et al. 2018; Murphy-Beiner and Soar 2020; Perkins et al. 2022) and thus represents an advancement of the literature as it will provide better knowledge of which acute effects may be more salutary for subacute effects. Yet, due to the small sample size the interaction models should be understood as exploratory. Finally, the current study addresses potential influence of participants’ past experiences and knowledge of psychedelics that may have influenced the acute and subacute experiences of the ayahuasca retreat.

## Methods

### Ayahuasca retreat and participant recruitment

We recruited adult participants who had registered to participate in a 4-day ayahuasca retreat, after they had participated in a year-long therapist certification program that covered topics such as relation between emotional problems, physical disease and treatment along with practical training (Butto 2019a, b, 2020). The program also provided participants with a common understanding of the potential effects of ayahuasca, strategies for navigating the psychedelic experience, and foster a mindset of openness, curiosity, and self-reflection. This type of preparation for psychedelic experiences (often referred to as “set”) (Hartogsohn 2017) is thought to be important for safety and salutary benefits of psychedelic experiences and the standardized approach to preparation is in line with recommendations for the safe and effective use of psychedelics in research and clinical settings (Johnson et al. 2008).

A phone screening was conducted to assess inclusion (age 18 or above and possession of a smartphone) and exclusion (recent or planned psychedelic use within two weeks of the retreat) criteria. Written informed consent was provided before participation in the study. The study received human subjects research ethics approval from the institutional review board of the Faculty of Social Welfare & Health Sciences, University of Haifa [#214/22].

### Procedure

First, participants completed an online baseline (pre-retreat) survey on their computers assessing sociodemographic background and past ayahuasca or other classic psychedelic use. Within one week prior to the retreat, participants reported on momentary affect and mindfulness skills, at three fixed times/day (09:00, 14:00, 19:00) over the course of five days on their mobile phones. Daily Qualtrics EMA survey links were sent to smartphones by WhatsApp messages. Participants then participated in the 4-day ayahuasca retreat, which employed a modern, integrative approach departing from traditional shamanic practices. The retreat was led by the course instructor and one helper, who were responsible for participant safety and support. This approach focused on applying integrative principles to help participants identify and resolve conflicts, emphasizing mental, emotional, and physical well-being. Each day was dedicated to one of four phases: excitation, expansion, contraction, and relaxation. Ceremonies were initiated at 9 pm and lasted 6 h, with participants having the opportunity to drink ayahuasca one to three times during each night. The ceremonies included music, guided meditations, and interfaith elements to facilitate participants’ journeys through these phases. Morning sessions allowed for sharing and integration of experiences, while afternoons involved ceremony preparation through meditation and breathing exercises. This structure aimed to create a comprehensive healing experience that aligned with contemporary therapeutic practices while honoring the sacredness of the ayahuasca journey.

**Fig. 1:**
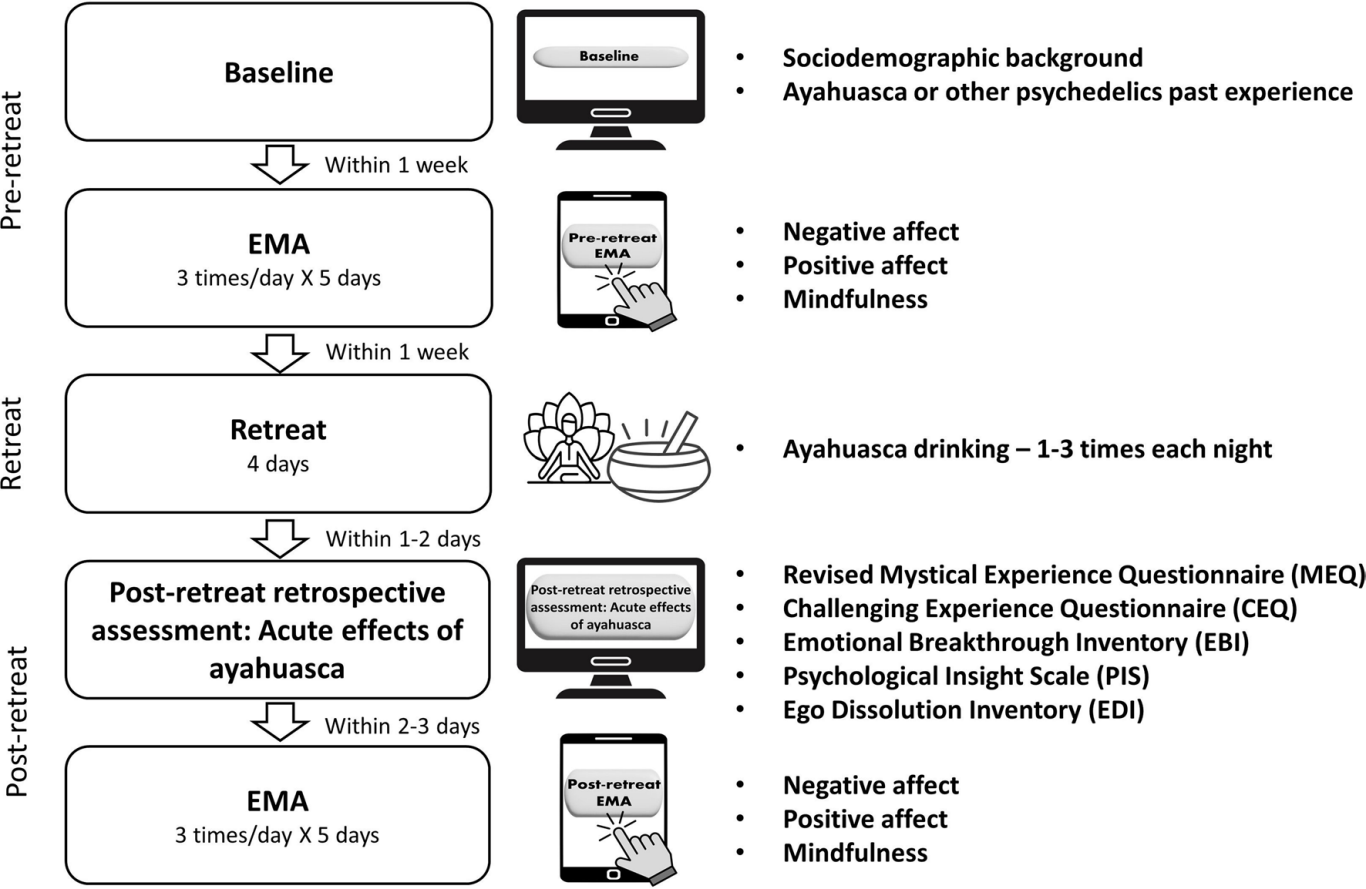
Study timeline

Then, 1–2 days following the retreat, participants completed a post-retreat online questionnaire on acute experiences induced by ayahuasca on their computers. Within 2–3 days after completing the post-retreat retrospective online questionnaire, participants reported on momentary affect and mindfulness skills, three times/day (morning, midday, evening) over the course of five days on their mobile phones. Figure 1 shows the study timeline.

Participants received email notifications for each EMA survey, with research staff following up on missed responses. Researchers’ involvement was limited to data collection procedures and coordinating study logistics with facilitators, without participating in ceremonies or sharing personal ayahuasca experiences. Participants were invited to provide feedback post-retreat; one participant’s concern about support during challenging experiences was noted but could not be independently verified (see supplement file for details). The researchers’ involvement with study participants was minimal, focusing solely on data collection aspects such as contacting participants, explaining study procedures, and ensuring adherence to the data collection schedule. The researchers were not involved in the ayahuasca ceremonies, which were conducted by experienced facilitators. Interaction between researchers and facilitators was also limited to coordinating study logistics and the researchers did not share their personal relationships to Ayahuasca with participants.

**Fig. 2:**
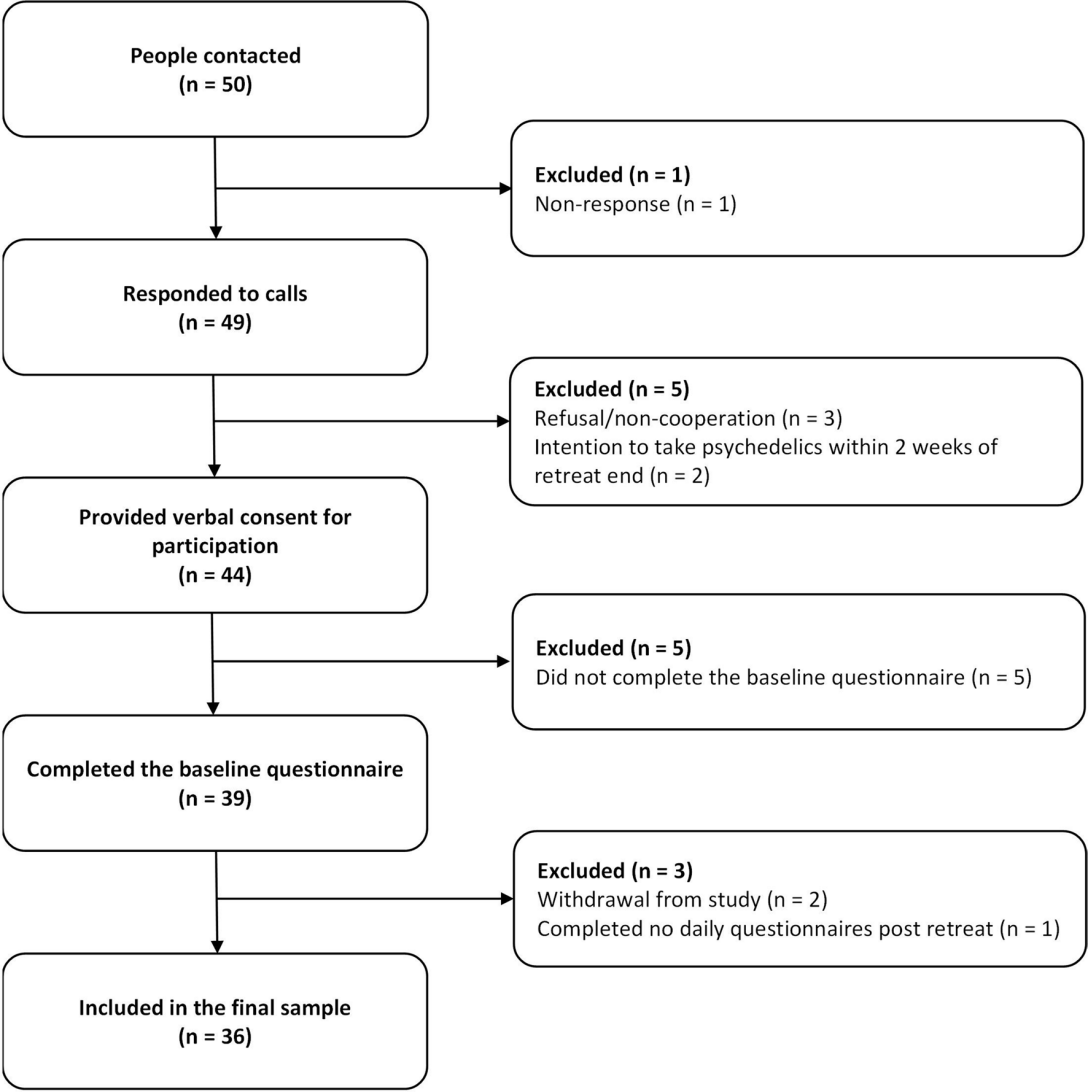
Recruitment and dropout flow chart

The final sample included 36 participants, sampled from two 4-day retreats. See Fig. 2 for the sample flow chart. Out of 1080 possible EMA surveys, 936 (86.60%) were fully completed and included in the analyses, 452 out of 540 (83.70%) in the pre-retreat EMA phase and 484 out of 540 (89.63%) in the post-retreat EMA phase. Participants completed a mean of 26.03 EMA assessments (*sd* = 3.10, range: 20–30). They missed fewer prompts in the post-retreat EMA (β = 2.44 (*sd* = 2.45)) compared to the pre-retreat EMA phase (β = 1.67 (*sd* = 1.66)) (β **=** -0.38, *p* = 0.02). There were no differences in response rates between the first and second halves of each EMA phase, or across variables such as sex, age, socioeconomic status, psychedelic experience and knowledge. One retreat group included 30 Israeli participants, of which 23 participated in the study, and a second retreat group included 20 European participants, of which 13 participated in the study. The retreat location, staff and program were the same. Reimbursement for participation was provided to the Israeli group, while the European group were volunteers. Detailed information on group similarity by background and outcome variables can be found in supplemental Table S1 and S2.

### Measures

#### Pre-retreat baseline retrospective assessment: background

At baseline, participant *demographics*, *past ayahuasca* and other *psychedelic use*, were assessed (0 = no lifetime use, 1 = lifetime use). *Psychedelic experiences and knowledge* were further assessed on a scale of 1 (disagree) to 5 (agree) (“I have extensive experience with psychedelic drugs”, “I possess advanced knowledge about psychedelics”) (adopted from Haijen et al. 2018).

#### Post-retreat retrospective assessment: Acute effects of Ayahuasca

*The Revised Mystical Experience Questionnaire (MEQ)*, a 30-item self-report items, measured mystical experiences linked to the retreat ayahuasca use (MacLean et al. 2012; Barrett et al. 2015). *The Challenging Experience Questionnaire (CEQ)*, a 26-item self-report tool, assessed challenging experiences during psychedelic use (Barrett et al. 2016). The 6-item self-reported *Emotional Breakthrough Inventory (EBI)* assessed emotional release/breakthrough linked to psychedelic use (Roseman et al. 2019). *The Ego Dissolution Inventory (EDI)*, an 8-item self-report questionnaire, measured ego-dissolution experiences related to the ayahuasca retreat (Nour et al. 2016).

#### Ecological momentary assessment: Subacute effects of Ayahuasca in daily living

An adapted version of the *Five Facet Mindfulness Questionnaire (FFMQ)* (Baer et al. 2006) was used to measure mindfulness skills in daily living (Snippe et al. 2015). Two FFMQ items were selected, for each facet based on robust subscale loadings, excluding the description factor that previous work indicated was not related to ayahuasca use (Murphy-Beiner and Soar 2020). *The International Positive and Negative Affect Schedule Short Form (I-PANAS-SF)* (Thompson 2007) was administered to assess negative and positive affect in daily living, including five positive emotions (alert, inspired, determined, attentive, active) and five negative emotions (upset, hostile, ashamed, tense, scared). All outcome and moderator variables were standardized by transforming each original variable into a new variable with a mean of 0 and a standard deviation of 1. See supplemental materials Table S3 and S4 for further details on the measures.

### Statistical analysis

Linear mixed effects models were used to test changes in affect and mindfulness skills in daily living from pre-to-post ayahuasca retreat (see supplement files for model equations). Models included random intercepts to account for subject-level clustering and per-participant autocorrelated (order 1) error structures for timepoints since first momentary assessment. Missingness was minor, with only ‘active’ and ‘scared’ items of the I-PANAS-SF skipped in three and two observations respectively, with one participant skipping both items in the same observation. Given the small number of missing items, we ran the model with the full information maximum likelihood (FIML), a likelihood-based approach to treating missing values (Cham et al. 2017). We applied robust standard error assessment using the rescaled maximum likelihood (MLR; Muthén and Satorra 1995), available in Mplus (Muthén and Muthén 2017).

To examine the main effect of the ayahuasca retreat on affect and mindfulness skills, models were run separately for positive affect, negative affect and mindfulness skills as dependent variables. Level 1 variables (post vs. pre-retreat, study time trend, morning/noon vs. evening) and Level 2 variables (age, male, previous ayahuasca experience/knowledge) were entered as covariates.

Additional models with interactive effects were run to examine whether acute psychedelic induced experiences and previous experience with and knowledge of psychedelic drugs predicted change in outcome variables from pre to post retreat. Specifically, these models were similar to the main models except we added interactions between the pre-post retreat variable and each of the different acute psychedelic experiences separately. To optimize statistical power and model parsimony, we simplified the interaction models excluding all predictors except from pre-post ayahuasca retreat indicator, linear time trend, moderator and the relevant interaction. The Benjamini Hochberg method was applied to correct for False Discovery Rate (FDR) (Benjamini and Hochberg 1995).

It is possible that some individuals show deterioration in outcome variables but that this is not detectable in the mixed effects models under an overall positive change in the sample. To zoom in on the potential of some individuals experiencing significant deterioration we computed a Reliable Change Index (RCI) for each outcome to compare the odds of deterioration from pre-to-post retreat in positive and negative affect, and mindfulness skills (Jacobson and Truax 1991). The standard error of difference was evaluated based on the baseline standard deviation (SD) of each scale and the test-retest reliability of each scale between pre- and post-retreat assessments. For each outcome, we calculated the percentage of participants who surpassed both a conservative 95% RCI confidence threshold for deterioration (1.96 RCI cutoff) and a more lenient 80% RCI confidence threshold for deterioration (0.84 RCI cutoff, as proposed by Wise 2004), to protect against the underestimation of deterioration associated with participation in the ayahuasca retreats.

## Results

### Descriptive results

The sample consisted of 19 men and 17 women (M(SD)age = 43.31(10.77). Nine of the 36 participants (25%) reported previous lifetime ayahuasca use and 26 (72%) reported other psychedelic lifetime use; rates similar to those reported in previous studies of ayahuasca use (Soler et al. 2016; Uthaug et al. 2018; van Oorsouw et al. 2022). See Table 1 for additional sample information.

**Table 1:** Demographic background of the sample (*n* = 36)

Variable	*N*	*%*	*Minimum*	*Maximum*
	*(Mean)*	*(SD)*		
**Gender**				
Men	19	52.78		
Women	17	47.22		
**Age**	(43.31)	(10.77)	24.00	74.00
**Marital status**				
Single	9	25		
Married	15	41.67		
Widowed	1	2.78		
Divorced	6	16.67		
Unmarried, domestic partnership	5	13.88		
**Children**				
Yes	21	58.33		
No	15	41.67		
**Monthly Income**				
Less than 1,450 Euro	5	13.89		
1,450–2,900 Euro	5	13.89		
2,900–4,350 Euro	8	22.22		
Above 4,350 Euro	14	38.89		
Prefer not to say	4	11.11		
**Employment** ^a^				
Full time	20	55.55		
Part-time	11	30.55		
Homemaker	1	2.78		
Retired	3	8.33		
Unemployed	2	5.55		
Student	2	5.55		
**Education**				
High school	4	11.11		
Post-secondary school	4	11.11		
Bachelor’s degree	15	41.67		
Master’s degree	13	36.11		
**Religion**				
Jewish	17	47.22		
Christian	10	27.78		
Muslim	4	11.11		
Other	5	13.89		
**Lifetime ayahuasca use**	9	25		
**Lifetime psychedelic use (non-Ayahuasca)**	26	72.2		
**Past psychedelic experience**	(2.49)	(1.33)	1.00	5.00
**Mystical Experiences Questionnaire**				
MEQ-30 mystical	(4.39)	(0.63)	1.73	5.00
MEQ-30 positive mood	(4.36)	(0.72)	1.33	5.00
MEQ-30 transcend time/space	(4.14)	(0.82)	2.00	5.00
MEQ-30 ineffable	(4.46)	(0.73)	2.33	5.00
MEQ-30 total score	(4.34)	(0.64)	1.97	5.00
**Challenging Experience Questionnaire**				
CEQ Grief/sadness	(0.37)	(0.25)	0.00	1.00
CEQ death	(0.32)	(0.35)	0.00	1.00
CEQ insanity	(0.25)	(0.26)	0.00	1.00
CEQ isolation	(0.13)	(0.17)	0.00	0.67
CEQ physical suffering	(0.47)	(0.20)	0.12	0.88
CEQ fear	(0.37)	(0.33)	0.00	1.00
CEQ total score	(0.32)	(0.20)	0.02	0.73
**Ego dissolution inventory**	(81.12)	(15.99)	27.43	100.00
**Emotional Breakthrough**	(83.90)	(21.29)	23.33	100.00
**EMA variables** ^**b**^				
Positive affect - Pre-retreat	(3.28)	(0.79)	1.00	3.80
Positive affect - Post-retreat	(3.77)	(0.71)	1.00	4.00
Negative affect - Pre-retreat	(1.45)	(0.57)	1.00	5.00
Negative affect - Post-retreat	(1.17)	(0.32)	1.00	5.00
Mindfulness - Pre-retreat	(4.65)	(0.85)	2.75	7.00
Mindfulness - Post-retreat	(5.47)	(0.76)	3.25	7.00

Note MEQ = Mystical Experience Questionnaire; CEQ = Challenging Experience Questionnaire; EMA = Ecological Momentary Assessment ^a^ Participants were able to choose more than one response. ^b^ Mean and standard deviations across individuals

### Mixed effect model results

**Fig. 3:**
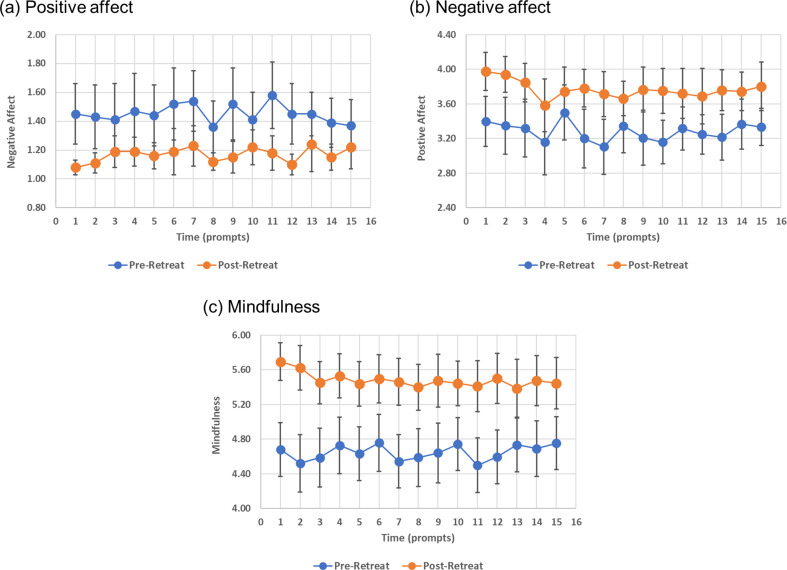
Means and confidence intervals of outcome variables pre- versus post-ayahuasca retreat. The blue line indicates pre-retreat mean scores at each prompt, and the orange line indicates post-retreat mean scores at each prompt. Vertical lines represent the 95% confidence intervals. (**a**) Negative affect. (**b**) Positive affect. (**c**) Mindfulness

Participants reported higher positive affect (β = 0. 681, *p* < 0.001), lower negative affect (β = -0. 614, *p* < 0.001), and higher mindfulness skills scores (β = 0. 939, *p* < 0.001) at post- relative to pre-retreat (see Table 2). Figure 3 shows the time trends, including means and confidence intervals, of the outcome variables at each prompt before and after the ayahuasca retreat (for further visual representation of data see S1 Spaghetti plots of individual and mean linear trajectories and regression lines). The only significant covariate was age, wherein older respondents had higher levels of mindfulness (β **=** 0. 015, *p* = 0.006).

**Table 2:** Results from mixed effects models predicting affect and mindfulness capabilities in daily life before and after the ayahuasca retreat (*n* = 36)

	Positive affect						Negative affect						Mindfulness					
	Model 1 A (without interactions)						Model 2 A (without interactions)						Model 3 A (without interactions)					
	Coef.	SE	z	*p*	95% CI		Coef.	SE	z	*p*	95% CI		Coef.	SE	z	*p*	95% CI	
Male	0.047	0.249	0.188	0.851	-0.442	0.535	0.376	0.197	1.906	0.057	-0.011	0.763	-0.254	0.223	-1.139	0.255	-0.691	0.183
Age	0.002	0.008	0.269	0.788	-0.013	0.018	-0.012	0.010	-1.188	0.235	-0.031	0.008	**0.015**	**0.005**	**2.742**	**0.006**	**0.004**	**0.025**
Survey time of the day (evening ref cat)																		
Morning	-0.051	0.065	-0.780	0.436	-0.178	0.077	0.037	0.050	0.742	0.458	-0.061	0.136	-0.003	0.043	-0.077	0.938	-0.087	0.081
Afternoon	0.076	0.050	1.527	0.127	-0.021	0.173	-0.045	0.068	-0.652	0.514	-0.179	0.089	-0.029	0.035	-0.820	0.412	-0.097	0.040
Study time trend	-0.004	0.007	-0.581	0.561	-0.017	0.009	0.002	0.006	0.321	0.748	-0.010	0.014	-0.003	0.006	-0.501	0.616	-0.014	0.008
Previous ayahuasca experience/knowledge	0.154	0.135	1.142	0.253	-0.110	0.419	-0.070	0.081	-0.865	0.387	-0.229	0.089	0.116	0.096	1.205	0.228	-0.073	0.305
Post-retreat (vs. pre-retreat)	**0.681**	**0.130**	**5.248**	**0.000**	**0.426**	**0.935**	**-0.614**	**0.144**	**-4.257**	**0.000**	**-0.897**	**-0.331**	**0.939**	**0.141**	**6.671**	**0.000**	**0.663**	**1.214**
Intercept	-0.372	0.306	-1.214	0.225	-0.972	0.228	0.400	0.284	1.410	0.158	-0.156	0.957	**-0.676**	**0.212**	**-3.192**	**0.001**	**-1.092**	**-0.261**

*Note* Coef. = coefficient; SE = standard error; CI = confidence interval. The significant variables are highlighted in bold

Interaction analyses (Table 3) documented that participants who experienced higher, relative to lower, levels of transcendence of time/space (β = 0. 160, *p* = 0.008, *pFDR* = 0.013), challenging experiences related to grief (β = 0. 165, *p* = 0.007, *pFDR* = 0.010), isolation (β = 0. 192, *p* = 0.002, *pFDR* = 0.003), physical suffering (β = 0. 148, *p* = 0.016, *pFDR* = 0.020), and total challenging experiences (β = 0. 155, *p* = 0.011, *pFDR* = 0.017), and emotional breakthrough (β = 0. 170, *p* = 0.005, *pFDR* = 0.007) during the ayahuasca retreat reported higher positive affect in daily living post-retreat. After adjustment for False Discovery Rate none of the ayahuasca-related experiences predicted post-retreat levels of negative affect or mindfulness in daily living. Finally, previous psychedelic experience/knowledge was not associated with the observed pre-to-post retreat changes in affect or mindfulness skills in daily living.

**Table 3:** Results from mixed effects models showing the degree to which moderators influence the effect of pre to post retreat outcomes—interaction coefficients

	Positive affect							Negative affect							Mindfulness						
	Model 1B moderators							Model 2B moderators							Model 3B moderators						
	Coef.	SE	z	*p*	95% CI		pFDR	Coef.	SE	z	*p*	95% CI		pFDR	Coef.	SE	z	*p*	95% CI		pFDR
MEQ-30:																					
Mystical	0.079	0.061	1.280	0.200	-0.042	0.199	0.040	-0.126	0.065	-1.930	0.053	-0.254	0.002	0.003	0.054	0.062	0.880	0.379	-0.067	0.175	0.027
Positive mood	0.099	0.062	1.610	0.108	-0.022	0.220	0.037	-0.084	0.066	-1.280	0.200	-0.212	0.045	0.020	0.069	0.062	1.120	0.264	-0.052	0.191	0.020
Transcend time/space	**0.160**	**0.060**	**2.660**	**0.008**	**0.042**	**0.277**	**0.013**	-0.117	0.065	-1.810	0.070	-0.244	0.010	0.010	0.127	0.060	2.090	0.036	0.008	0.245	0.007
Ineffable	0.006	0.119	0.050	0.962	-0.228	0.239	0.050	-0.115	0.065	-1.770	0.077	-0.243	0.013	0.013	0.073	0.061	1.180	0.238	-0.048	0.193	0.017
Total	0.113	0.061	1.850	0.064	-0.007	0.233	0.027	-0.124	0.065	-1.900	0.057	-0.251	0.004	0.007	0.082	0.061	1.340	0.179	-0.038	0.203	0.013
CEQ:																					
Grief/sadness	**0.165**	**0.061**	**2.680**	**0.007**	**0.044**	**0.285**	**0.010**	-0.023	0.066	-0.350	0.726	-0.153	0.107	0.040	0.030	0.063	0.480	0.632	-0.094	0.154	0.043
Death	0.038	0.062	0.610	0.544	-0.084	0.159	0.047	0.102	0.065	1.560	0.118	-0.026	0.230	0.017	-0.053	0.062	-0.860	0.390	-0.174	0.068	0.030
Insanity	0.132	0.062	2.150	0.032	0.011	0.253	0.023	0.014	0.066	0.210	0.830	-0.115	0.143	0.043	0.064	0.062	1.040	0.299	-0.057	0.185	0.023
Isolation	**0.192**	**0.061**	**3.140**	**0.002**	**0.072**	**0.312**	**0.003**	-0.076	0.066	-1.160	0.248	-0.205	0.053	0.023	0.047	0.063	0.750	0.454	-0.076	0.170	0.033
Physical suffering	**0.148**	**0.061**	**2.410**	**0.016**	**0.028**	**0.268**	**0.020**	0.035	0.066	0.530	0.596	-0.094	0.164	0.037	0.007	0.062	0.120	0.905	-0.115	0.130	0.050
Fear	0.109	0.062	1.760	0.079	-0.012	0.230	0.033	0.011	0.066	0.170	0.864	-0.118	0.141	0.047	0.084	0.062	1.350	0.178	-0.038	0.205	0.010
Total	**0.155**	**0.061**	**2.530**	**0.011**	**0.035**	**0.275**	**0.017**	0.011	0.066	0.170	0.865	-0.118	0.140	0.050	0.046	0.062	0.740	0.462	-0.076	0.167	0.037
Ego dissolution	0.111	0.061	1.820	0.069	-0.009	0.231	0.030	-0.036	0.066	-0.550	0.579	-0.165	0.092	0.033	0.032	0.062	0.520	0.600	-0.089	0.153	0.040
Emo. breakthrough	**0.170**	**0.061**	**2.800**	**0.005**	**0.051**	**0.289**	**0.007**	-0.072	0.065	-1.100	0.270	-0.200	0.056	0.027	0.174	0.061	2.830	0.005	0.054	0.294	0.003
Past psych. experience	-0.051	0.062	-0.830	0.408	-0.172	0.070	0.043	-0.039	0.066	-0.590	0.552	-0.168	0.090	0.030	0.019	0.062	0.300	0.765	-0.103	0.140	0.047

*Note* MEQ-30 = Mystical Experience Questionnaire; CEQ = Challenging Experience Questionnaire; Emo. breakthrough = Emotional breakthrough; Past psych. experience = Past psychedelic experience; Coef. = coefficient; SE = standard error; CI = confidence interval; pFDR = False Discovery Rate (FDR) adjusted p values. The significant variables based on pFDR are highlighted in bold. The rejection criteria was alpha = 0.05 adjusted for FDR by the Benjamini-Hochberg method (1995)

In terms of RCI, none of the retreat participants showed evidence of statistically reliable deterioration at the 95% RCI confidence level for any of the outcomes. One person showed reliable deterioration at the 80% RCI level for positive affect, and another person showed reliable deterioration at this RCI level for negative affect. No participant showed reliable deterioration at 80% RCI level for mindfulness skills.

## Discussion

The current study contributes to the growing body of literature on the subacute effects of ayahuasca by employing an EMA approach, which offers several advantages over traditional retrospective self-report measures used in previous research. By assessing affect and mindfulness skills multiple times a day, over several days before and after the retreat, we were able to capture participants’ experiences closer to their occurrence in their natural environment. This approach reduces the reliance on retrospective recall, which may be subject to bias and influenced by current mood states (Hektner et al. 2007; Shiffman et al. 2008; Carlson et al. 2016), and provides a more ecologically valid representation of participants’ daily lives.

Results show that, as predicted, ayahuasca had a significant salutary impact on subacute levels of positive and negative affect (Perkins et al. 2022) as well as mindfulness skills (Thomas et al. 2013; Soler et al. 2016; van Oorsouw et al. 2022) in daily living, well beyond the acute time course of ayahuasca use. These ayahuasca-related effects did not depend on past psychedelic experiences or knowledge related to psychedelics. Results are consistent with recent studies of ayahuasca which also documented salutary properties and potentially therapeutic applications of its use (Domínguez-Clavé et al. 2016; Agin-Liebes et al. 2022).

Another key strength of our study is the comprehensive assessment of a wide range of acute psychedelic experiences, including mystical experiences, challenging experiences, ego dissolution, and emotional breakthrough. By examining this broad spectrum of acute effects, we were able to identify specific dimensions that predict subacute outcomes. This approach provides a nuanced understanding of the complex nature of ayahuasca experiences and their potential impact on individuals’ daily lives.

Our findings indicate that several dimensions of acute challenging experiences induced by ayahuasca were prospectively associated with gains in subacute positive affect in daily living. These include acute challenging experiences related to grief/sadness, isolation, physical suffering, and total challenging experiences. While these results suggest potentially paradoxical salutary effects of challenging experiences, it is crucial to interpret them cautiously, considering the complexities and contradictions present in the existing literature. The relationship between challenging psychedelic experiences and subsequent outcomes is complex and not uniformly positive. While observational and retrospective studies have suggested potential benefits of challenging experiences (Barrett et al. 2016; Carbonaro et al. 2016; Johnstad 2021; Weiss et al. 2021; Gashi et al. 2021; Bouso et al. 2022; Lake and Lucas 2023), a double-blind, randomized, controlled trial recently failed to find positive associations between challenging experiences and improved outcomes (Weiss et al. 2024). Moreover, challenging experiences has been associated with post-acute distress, functional impairment, and the need for medical attention (Larsen 2016; Durante et al. 2020; Barber et al. 2022; Bouso et al. 2022; Simonsson et al. 2023; Bremler et al. 2023), with rare cases resulting in psychiatric complications or harm to self or others (Carbonaro et al. 2016; Zeifman et al. 2021; Bremler et al. 2023).

The discrepancy in research may be attributed to several factors and needs further investigation. For instance, the setting and population may play a crucial role. Our sample consisted of non-clinical participants in a safe, controlled environment with proper preparation. This context might facilitate the integration of challenging experiences into positive outcomes, unlike studies conducted in less controlled settings or with clinical populations (Larsen 2016; Carbonaro et al. 2016; Barber et al. 2022; Bremler et al. 2023). Future research should examine the role of integration in the effect of challenging experiences on well-being changes after psychedelic use. It is also worth noting that our study’s small sample size limits the reliability and generalizability of our findings. The associations we observed between challenging experiences and positive affect require replication in larger, more diverse samples.

If replicated in future research, the concept of “post-traumatic growth” provides a potential framework for understanding how challenging experiences might lead to positive outcomes (Tedeschi and Calhoun 2004). In the context of ayahuasca use, challenging experiences may serve as opportunities for individuals to confront and process difficult emotions, memories, or aspects of their lives, leading to increased self-awareness, emotional resilience, and psychological growth (Belser et al. 2017; Lafrance et al. 2017). This highlights the importance of integrating and making meaning of challenging experiences during ayahuasca use, as they may contribute to positive subacute outcomes.

Future research could aim to clarify the conditions under which challenging ayahuasca experiences may lead to positive outcomes. This could include investigating the role of set and setting, preparation and integration practices, and individual differences in responding to challenging experiences. Longitudinal studies with larger sample sizes are needed to establish more reliable causal relationships between acute challenging experiences and long-term outcomes.

Inconsistent with our prediction, none of the measured acute (psychedelic) experiences induced by ayahuasca were related to subacute changes in negative affect or mindfulness in daily living beyond the retreat. Notably, one previous study also failed to find an association between acute ayahuasca use experiences and subacute effects on negative affect (Agin-Liebes et al. 2022). We speculate that, if replicable and robust, the observed specificity of acute effects induced by ayahuasca use on subacute positive, but not negative affect or mindfulness in daily living, merits ongoing scientific study. For example, it is possible that our findings are specific to non-clinical samples, and that moderation by acute psychedelic experiences are evident in clinical populations suffering from mood disorders. Furthermore, additional experiences induced by ayahuasca, not assessed here, may be important to its effects on negative affect and mindfulness. These may include connectedness, altered self-perceptions, and expanded emotional spectrum (Breeksema et al. 2020). Alternatively, it is possible that observed benefits of ayahuasca use for negative affect and mindfulness may, in fact, be artifactual or secondary to regression to the mean or other expectancy effects. It is important that the observed robust differential pattern of salutary effects on positive vs. negative affect and mindfulness, is replicated, in future prospective and randomized study designs.

It is important to note that our study, with measurements up to 5 days post-retreat, focuses on what we term ‘subacute’ effects. However, the distinction between subacute and longer-term effects is not firmly established in psychedelic research. Future studies with extended follow-up periods are needed to clarify the trajectory of ayahuasca’s effects over time and to determine whether subacute and long-term effects represent distinct phases. This distinction is crucial for understanding the full temporal profile of ayahuasca’s effects.

Critically, none of the participants demonstrated significant clinical deterioration in any of the measured subacute outcomes post-retreat, when applying the traditional 95% confidence interval. Only 5.5% of participants (*n* = 2) may have experienced significant deterioration in either positive or negative affect, when applying the more liberal 80% confidence interval threshold. Notably, it is possible, if not likely, that a similar or even greater % of participants would demonstrate similar deterioration in these outcomes in daily living, even in the absence of ayahuasca (Hadash et al. 2024). The low incidence of adverse effects may be attributed to careful preparation and standardized set and setting. Participants completed a year-long therapist certification program before the retreat, covering relevant topics and practical training (Butto 2019a, b, 2020). This preparation, along with the guided, safe setting led by experienced staff, likely contributed to positive outcomes, aligning with established literature on the importance of set and setting in psychedelic experiences (Johnson et al. 2008; Hartogsohn 2017). However, one participant’s feedback highlighted concerns about inadequate management of challenging experiences during the ceremonies, underscoring the need for adequately trained staff and clear protocols in psychedelic retreat settings (Sznitman et al. 2024). Future research should examine best practices for ensuring participant safety and wellbeing in naturalistic ayahuasca contexts.

The study is limited in a number of important ways that may qualify findings and inform future studies. First, we did not control or randomize participants to ayahuasca use or dose. Although such a design is extremely complicated in the field of psychedelics due to blinding-failures (Muthukumaraswamy et al. 2021), it is important for causal inference and to rule out placebo and expectancy effects. Second, a significantly larger sample size may be needed to examine robust estimates, and potential predictors of or conditions for, possible adverse effects of ayahuasca use. Furthermore, while our comprehensive assessment of acute psychedelic experiences provides valuable insights, the exploratory nature of our interaction analyses, combined with our modest sample size, necessitates cautious interpretation. The breadth of acute factors examined, while an advancement relative to extant knowledge, introduces challenges related to multiple comparisons and statistical power. To address these challenges, we implemented the Benjamini-Hochberg method for FDR correction. Despite these measures, these exploratory findings should be viewed primarily as hypothesis-generating for future research with larger samples, rather than as definitive conclusions. The trade-off between comprehensiveness and statistical power in this study underscores the need for larger-scale investigations to more robustly examine the relationships between specific acute experiences and subacute outcomes. While our approach to controlling for multiple testing strengthens the reliability of our findings, the limitations imposed by our sample size remain an important consideration in interpreting the results. Third, although it is scientifically plausible to implicate ayahuasca in the observed effects, the study design cannot tease apart the role of ayahuasca, from various features of this particular 4-day retreat, or preparation pre-retreat. Fourth, measurement was limited to numerical self-reports and acute effects were measured 1–2 days after the ayahuasca retreat. Real-time assessments were deemed unfeasible as they could interfere with acute effects. Yet, future study could benefit from additional methods, including real-time assessments, structured or phenomenological interviews, second-persons observations, and behavioral assessments (Hadash and Bernstein 2019; Petitmengin et al. 2019; Hadash et al. 2023). Fifth, while this study used validated scales of acute psychedelic experiences, the definition and measurement of these constructs have been criticized for being conceptually vague and overlapping (Taves 2020; Canby et al. 2024). Work is being done to improve measurements of these experiences which will be useful for furthering this field of research in the future (Canby et al. 2024). Sixth, although the study may have implications for therapeutic benefits of ayahuasca use, it is important that similar data are collected among selected or clinical populations who may respond differently to acute effects of ayahuasca or demonstrate different subacute effects of use. Finally, the generalizability of findings to other settings and participant expectancies may be limited by the naturalistic design and self-selected sampling.

A 4-day ayahuasca retreat related to subacute improvement on positive and negative affect as well as greater mindfulness skills in daily living. Moreover, key acute experiences induced by ayahuasca, including emotional breakthrough, transcendence of time/space, and various challenging experiences, may have a mechanistic role in key salutary properties of ayahuasca use, most notably for positive affect.

## Electronic supplementary material

Below is the link to the electronic supplementary material.

Supplementary Material 1

## Data Availability

Data is available upon request, but due to the sensitivity of the data it will not be released in full. Furthermore, the research team is still working on additional analyses.
