# Supplementary material for: A prospective ecological momentary assessment study of an ayahuasca retreat: exploring the salutary impact of acute psychedelic experiences on subacute affect and mindfulness skills in daily life
Source: Psychopharmacology (Berl). 2025 Jan 18;242(3):545–61. doi: 10.1007/s00213-024-06704-8 (PMC11861408; doi:10.1007/s00213-024-06704-8)
Supplement: Supplementary file 1 — Supplementary Material 1 [file 213_2024_6704_MOESM1_ESM.docx]

**Title: A Prospective Ecological Momentary Assessment Study of an Ayahuasca Retreat: Exploring the Salutary Impact of Acute Psychedelic Experiences on Subacute Affect and Mindfulness Skills in Daily Life**

**Journal name: Psychopharmacology**

**Author names:** Sznitman, S. R.*, Behar, Y., Dicker-Oren, S. D., Shochat, T., Meiri, D., Butto, N., Roe, D., & Bernstein, A.

* Corresponding author - School of Public Health, University of Haifa, Israel. Address: 199 Aba Khoushy Ave., Mount Carmel, Haifa, Israel. P.O.Box: 3338. ZIP: 3103301. Telephone number: +97248288604. Email: [sznitman@research.haifa.ac.il](mailto:sznitman@research.haifa.ac.il); sznitmans@gmail.com

**Table S1** Demographic background and moderator variables of the sample by Israel-Europe groups

|  | **Total (n = 36)** | | **Israel (n = 23)** | | **Europe (n = 13)** | |  |  |
| --- | --- | --- | --- | --- | --- | --- | --- | --- |
| **Variable** | ***N*** | ***%*** | ***N*** | ***%*** | ***N*** | ***%*** | ***Statistic*** | ***p*** |
|  | ***(Mean)*** | ***(SD)*** | ***(Mean)*** | ***(SD)*** | ***(Mean)*** | ***(SD)*** |  |  |
| **Gender** |  |  |  |  |  |  | 0.36 | 0.55 |
| Men | 19 | 52.78 | 13 | 56.52 | 6 | 46.15 |  |  |
| Women | 17 | 47.22 | 10 | 43.48 | 7 | 53.85 |  |  |
| **Age** | ((43.31)) | ((10.77)) | ((44.65)) | ((12.25)) | ((40.92)) | ((7.33)) | 122.50 | 0.37 |
| **Marital status** |  |  |  |  |  |  | 3.11 | 0.54 |
| Single | 9 | 25 | 5 | 21.74 | 4 | 30.77 |  |  |
| Married | 15 | 41.67 | 10 | 43.48 | 5 | 38.46 |  |  |
| Widowed | 1 | 2.78 | 1 | 4.35 | 0 | 0.00 |  |  |
| Divorced | 6 | 16.67 | 5 | 21.74 | 1 | 7.69 |  |  |
| Unmarried, domestic partnership | 5 | 13.88 | 2 | 8.70 | 3 | 23.08 |  |  |
| **Children** |  |  |  |  |  |  | 3.31 | 0.07 |
| Yes | 21 | 58.33 | 16 | 69.57 | 5 | 38.46 |  |  |
| No | 15 | 41.67 | 7 | 30.43 | 8 | 61.54 |  |  |
| **Monthly Income** |  |  |  |  |  |  | 7.16 | 0.13 |
| Less than 1,450 Euro | 5 | 13.89 | 1 | 4.35 | 4 | 30.77 |  |  |
| 1,450 - 2,900 Euro | 5 | 13.89 | 4 | 17.39 | 1 | 7.69 |  |  |
| 2,900 - 4,350 Euro | 8 | 22.22 | 7 | 30.43 | 1 | 7.69 |  |  |
| Above 4,350 Euro | 14 | 38.89 | 8 | 34.78 | 6 | 46.15 |  |  |
| Prefer not to say | 4 | 11.11 | 3 | 13.04 | 1 | 7.69 |  |  |
| **Employment** ^a^ |  |  |  |  |  |  |  |  |
| Full time | 20 | 55.55 | 13 | 56.52 | 7 | 53.85 | 0.02 | 0.88 |
| Part-time | 11 | 30.55 | 9 | 39.10 | 2 | 15.40 | 3.42 | 0.26 |
| Homemaker/ Retired/ Unemployed | 6 | 16.66 | 3 | 13.04 | 3 | 23.08 | 0.51 | 0.65 |
| Student | 2 | 5.55 | 1 | 4.35 | 1 | 7.69 | 0.56 | 0.67 |
| **Education** |  |  |  |  |  |  | 1.05 | 0.79 |
| High school | 4 | 11.11 | 3 | 13.04 | 1 | 7.69 |  |  |
| Post-secondary school | 4 | 11.11 | 3 | 13.04 | 1 | 7.69 |  |  |
| Bachelor's degree | 15 | 41.67 | 10 | 43.48 | 5 | 38.46 |  |  |
| Master's degree | 13 | 36.11 | 7 | 30.43 | 6 | 46.15 |  |  |
| **Religion** |  |  |  |  |  |  | **18.88** | **<.001** |
| Jewish | 17 | 47.22 | 17 | 73.91 | 0 | 0.00 |  |  |
| Christian | 10 | 27.78 | 4 | 17.39 | 6 | 46.15 |  |  |
| Muslim | 4 | 11.11 | 1 | 4.35 | 3 | 23.08 |  |  |
| Other | 5 | 13.89 | 1 | 4.35 | 4 | 30.77 |  |  |
| **Lifetime ayahuasca use** | 9 | 25 | 4 | 17.39 | 5 | 38.46 | 0.35 | 0.24 |
| **Lifetime Psychedelic use (non-Ayahuasca)** | 26 | 72.2 | 17 | 73.91 | 9 | 69.23 | 1.25 | 0.76 |
| **MEQ-30:** |  |  |  |  |  |  |  |  |
| Mystical | (4.39) | (0.63) | (4.48) | (0.69) | (4.25) | (0.49) | 93.00 | 0.06 |
| Positive mood | (4.36) | (0.72) | (4.36) | (0.83) | (4.36) | (0.50) | 130.00 | 0.51 |
| Transcend time/space | (4.14) | (0.82) | (4.19) | (0.88) | (4.06) | (0.71) | 125.00 | 0.42 |
| Ineffable | (4.46) | (0.73) | (4.49) | (0.76) | (4.41) | (0.71) | 136.00 | 0.63 |
| Total | (4.34) | (0.64) | (4.40) | (0.71) | (4.25) | (0.52) | 108.50 | 0.18 |
| **CEQ:** |  |  |  |  |  |  |  |  |
| Grief/sadness | (0.37) | (0.25) | (0.37) | (0.25) | (0.38) | (0.26) | 146.50 | 0.92 |
| Death | (0.32) | (0.35) | (0.31) | (0.36) | (0.32) | (0.34) | 144.00 | 0.85 |
| Insanity | (0.25) | (0.26) | (0.21) | (0.25) | (0.31) | (0.28) | 116.00 | 0.26 |
| Isolation | (0.13) | (0.17) | (0.12) | (0.15) | (0.15) | (0.21) | 144.00 | 0.85 |
| Physical suffering | (0.47) | (0.20) | (0.47) | (0.19) | (0.48) | (0.24) | 148.50 | 0.97 |
| Fear | (0.37) | (0.33) | (0.35) | (0.34) | (0.41) | (0.33) | 133.50 | 0.60 |
| Total | (0.32) | (0.20) | (0.31) | (0.20) | (0.34) | (0.21) | 135.50 | 0.65 |
| **Ego dissolution** | (81.12) | (15.99) | (82.99) | (15.94) | (77.82) | (16.17) | 112.00 | 0.22 |
| **Emo. breakthrough** | (83.90) | (21.29) | (82.41) | (22.34) | (86.53) | (19.90) | 144.50 | 0.87 |
| **Past. psych. experience** | (2.49) | (1.33) | (2.48) | (1.46) | (2.50) | (1.12) | 145.50 | 0.89 |
| *Note*. MEQ-30 = Mystical Experience Questionnaire; CEQ = Challenging Experience Questionnaire; Emo. breakthrough = Emotional breakthrough; Past psych. experience = Past psychedelic experience; SD = standard deviation. Due to small cell sizes, Fisher's exact test was used to calculate p-values for categorical variables with two sub-categories. Monte Carlo simulation was used for categorical categories with more than two subcategories. The Mann-Whitney U test was conducted for numerical variables. Significant p values are highlighted in bold. ^a^ Participants were able to choose more than one response. | | | | | | | | |

**Table S2** RM-Anova of Israel-Europe comparison of negative affect, positive affect and mindfulness

|  | **Negative affect** | | | **Positive affect** | | | **Mindfulness** | | |
| --- | --- | --- | --- | --- | --- | --- | --- | --- | --- |
|  | ***Mean*** | ***SD*** |  | ***Mean*** | ***SD*** |  | ***Mean*** | ***SD*** |  |
| Israel Pre-retreat | 1.52 | 0.58 |  | 3.35 | 0.73 |  | 4.58 | 0.77 |  |
| Israel Post-retreat | 1.39 | 0.50 |  | 3.39 | 0.67 |  | 4.70 | 0.82 |  |
| Europe Pre-retreat | 1.11 | 0.17 |  | 4.01 | 0.57 |  | 5.64 | 0.65 |  |
| Europe Post-retreat | 1.24 | 0.43 |  | 3.80 | 0.81 |  | 5.43 | 0.83 |  |
|  | **F** | **p** | **η²p** | **F** | **p** | **η²p** | **F** | **p** | **η²p** |
| Within-subjects |  |  |  |  |  |  |  |  |  |
| Phase (Pre-retreat; Post-retreat) | **14.51** | **0.00** | **0.29** | **22.27** | **<.001** | **0.39** | **57.77** | **<.001** | **0.62** |
| Observation (First; Last) | 0.00 | 1.00 | 0.00 | 1.05 | 0.31 | 0.03 | 0.34 | 0.56 | 0.01 |
| Phase x Observation | 3.40 | 0.07 | 0.09 | 3.30 | 0.08 | 0.09 | 2.84 | 0.10 | 0.08 |
| Including region groups |  |  |  |  |  |  |  |  |  |
| Between-subjects |  |  |  |  |  |  |  |  |  |
| Region (Israel; Europe) | 0.37 | 0.55 | 0.01 | 0.24 | 0.63 | 0.01 | 1.82 | 0.19 | 0.05 |
| Within-subjects |  |  |  |  |  |  |  |  |  |
| Phase (Pre-retreat; Post-retreat) | **11.91** | **0.00** | **0.26** | **23.88** | **<.001** | **0.41** | **57.13** | **<.001** | **0.63** |
| Region x Phase | 0.41 | 0.53 | 0.01 | 1.39 | 0.25 | 0.04 | 0.92 | 0.35 | 0.03 |
| Observation (First; Last) | 0.02 | 0.90 | 0.00 | 1.22 | 0.28 | 0.04 | 0.38 | 0.54 | 0.01 |
| Region x Observation | 0.20 | 0.66 | 0.01 | 0.22 | 0.64 | 0.01 | 0.06 | 0.81 | 0.00 |
| Phase x Observation | 3.71 | 0.06 | 0.10 | 2.86 | 0.10 | 0.08 | 2.54 | 0.12 | 0.07 |
| Region x Phase x Observation | 0.37 | 0.55 | 0.01 | 0.01 | 0.92 | 0.00 | 0.00 | 0.99 | 0.00 |
| *Note*. SD = standard deviation; F = F-ratio; p = p-value; η²p = partial eta squared. Significant effects are highlighted in bold. | | | | | | | | | |

| **Table S3** Study measurements | | | |
| --- | --- | --- | --- |
| **Phase** | **Measure** | **Description** | **Reliability (α)** |
| **Baseline measures** | *Demographics background* | Age, gender, religion, employment, education level, marital, parenthood and financial status, past ayahuasca use and previous lifetime experience with psychedelics other than ayahuasca. | -- |
|  | *Potential bias in psychological expectancies versus pharmacological/ceremony effects on pre-to-post ayahuasca changes (Haijen, et al., 2018)* | "I have extensive experience with psychedelic drugs" and "I possess advanced knowledge about psychedelics" | -- |
| **Post-ceremony measures** | *The Revised Mystical Experience Questionnaire (MEQ; Barrett et al., 2015; MacLean et al., 2012*) | Mystical aspects of the ayahuasca experience | 0.97 for total score, and a range from 0.86 (Ineffability) to 0.95 (Mystical) |
|  | *The Challenging Experience Questionnaire (CEQ; Barrett, et al., 2016)* | Facets of challenging experiences during psychedelic use. | 0.94 for total score, and a range from 0.71 (physical distress) to 0.94 (fear). Paranoia factor had a low α (-0.11) and was omitted from analyses. |
|  | *The Emotional Breakthrough Inventory (EBI; Leor Roseman, et al., 2019)* | Emotional release/breakthrough linked to psychedelic experiences | 0.92 |
|  | *Psychological Insight Scale (PIS; Peill, et al., 2022)* | Psychological insight resulting from a psychedelic encounter | 0.96 |
|  | *The Ego Dissolution Inventory (EDI; Nour et al., 2016)* | Ego-dissolution experiences | 0.83 |
| **Daily measures** | *Adapted version of the Five Facet Mindfulness Questionnaire; excluding the description factor (FFMQ; Baer et al., 2006; Murphy-Beiner & Soar, 2020; Snippe et al., 2015)* | Momentary mindfulness assessment:  Since the last survey, to what extent have you:  1. Deliberately notice the sensations of your body moving when you were walking.  2. Paid attention to sounds, such as clocks ticking, birds chirping or cars passing.  3. Been "running on automatic" without much awareness of what you are doing. (reverse coded)  4. Rushed through activities without being really attentive to them (reverse coded).  5. Thought that some of your emotions are bad or inappropriate and that you shouldn't feel them (reverse coded).  6. Disapproved of yourself for having irrational or inappropriate thoughts (reverse coded)  7. Watched/observed your feelings without getting lost in them  8. Noticed and let go of distressing thoughts or images when you had them | 0.78 |
|  | *Positive and Negative Affect Schedule Short Form (I-PANAS-SF; Thompson, 2007)* | Momentary positive affect:  To what extent do you feel each of the following emotions right now?  1. Alert  2. Inspired  3. Determined  4. Attentive  5. Active | 0.83 |
|  |  | Momentary negative affect:  To what extent do you feel each of the following emotions right now?  1. Upset  2. Hostile  3. Ashamed  4. Tense  5. Scared | 0.79 |

**Table S4** Characteristics of outcome and moderator variables

|  | *Mean* | *SD* | Min. unst. | Max unst. | Min. stand. | Max stand. | Skewness | Kurtosis |
| --- | --- | --- | --- | --- | --- | --- | --- | --- |
| Outcome variables: |  |  |  |  |  |  |  |  |
| Negative affect | 1.31 | 0.48 | 1.00 | 4.00 | -0.64 | 5.62 | 2.47 | 6.88 |
| Positive affect | 3.53 | 0.79 | 1.00 | 5.00 | -3.21 | 1.86 | -0.49 | 0.16 |
| Mindfulness | 5.07 | 0.91 | 2.75 | 7.00 | -2.56 | 2.13 | -0.07 | -0.67 |
| Moderators: |  |  |  |  |  |  |  |  |
| MEQ-30: |  |  |  |  |  |  |  |  |
| Mystical | 4.39 | 0.63 | 1.73 | 5.00 | -4.25 | 0.96 | -2.40 | 8.33 |
| Positive mood | 4.36 | 0.72 | 1.33 | 5.00 | -4.20 | 0.88 | -2.19 | 7.69 |
| Transcend time/space | 4.14 | 0.82 | 2.00 | 5.00 | -2.72 | 1.05 | -1.08 | 0.62 |
| Ineffable | 4.46 | 0.73 | 2.33 | 5.00 | -2.97 | 0.73 | -1.54 | 1.93 |
| Total | 4.34 | 0.64 | 1.97 | 5.00 | -3.76 | 1.02 | -1.74 | 4.16 |
| CEQ: |  |  |  |  |  |  |  |  |
| Grief/sadness | 0.37 | 0.25 | 0.00 | 1.00 | -1.53 | 2.51 | 0.43 | -0.19 |
| Death | 0.32 | 0.35 | 0.00 | 1.00 | -0.93 | 2.00 | 0.84 | -0.62 |
| Insanity | 0.25 | 0.26 | 0.00 | 1.00 | -0.95 | 2.86 | 1.38 | 1.65 |
| Isolation | 0.13 | 0.17 | 0.00 | 0.67 | -0.81 | 3.18 | 1.41 | 1.82 |
| Physical suffering | 0.47 | 0.20 | 0.12 | 0.88 | -1.73 | 1.99 | 0.28 | -1.03 |
| Fear | 0.37 | 0.33 | 0.00 | 1.00 | -1.12 | 1.87 | 0.56 | -1.01 |
| Total | 0.32 | 0.20 | 0.02 | 0.73 | -1.51 | 2.04 | 0.39 | -0.88 |
| Ego dissolution | 81.12 | 15.99 | 27.43 | 100.00 | -3.41 | 1.17 | -1.61 | 2.84 |
| Emo. breakthrough | 83.90 | 21.29 | 23.33 | 100.00 | -2.89 | 0.76 | -1.83 | 2.61 |
| Past psych. experience | 2.49 | 1.33 | 1.00 | 5.00 | -1.14 | 1.94 | 0.23 | -1.31 |
| *Note*. MEQ-30 = Mystical Experience Questionnaire; CEQ = Challenging Experience Questionnaire; Emo. breakthrough = Emotional breakthrough; Past psych. experience = Past psychedelic experience; SD = standard deviation; Min. unst. = minimum value of unstandardized scores; Max. unst. = maximum value of unstandardized scores; Min. stand. = minimum value of standardized scores; Max. stand. = maximum value of standardized scores. | | | | | | | | |

**Model equations:**

Level-1 Model

y_it_ (standardized positive/negative affect, mindfulness outcome score_ij_) =

β_0j_ + β_1_*(post vs. pre ceremony_ij_) + β_2_*(study time trend_ij_) + β_3_*(morning/noon vs. evening_ij_) + r_ij_, Note, y_it_ was tested in its standardized form, as written above; and β_1,_ β_2,_ and β_3_ are fixed.

Level-2 Model

β_0j_ = γ_00_ + γ_01_*(age_j_) + γ_02_*(male vs. female_j_) + γ_03_*(previous ayahuasca experience/knowledge_j_) + u_0j;_

Combined Model

y_it_ (standardized positive/negative affect, mindfulness outcome score_ij_) =

γ_00_ + γ_01_*(age_i_) + γ_02_*(male vs. female_i_) + γ_03_*(previous ayahuasca experience/knowledge_i_) + β_1_*(post vs. pre ceremony_i_ + β_2_*(study time trend_it_) + β_3_*(morning/noon vs. evening_it_) + u_0i_+ r_it_

Where i is the index of individual respondents (1,2,…,36), and t is an index of time (1,2,..,30).

**Post-retreat feedback:**

One participant contacted the research team with concerns about how a particular challenging situation was handled during the retreat. The participant felt that another attendee was not receiving adequate support from the organizers and helpers. While this feedback is important to note, it represents a single participant's perspective and could not be independently verified. The research team offered to facilitate communication with the organizers, but the participant declined. This incident underscores the complexity of conducting research in naturalistic settings and the need for clear safety protocols in psychedelic retreats.


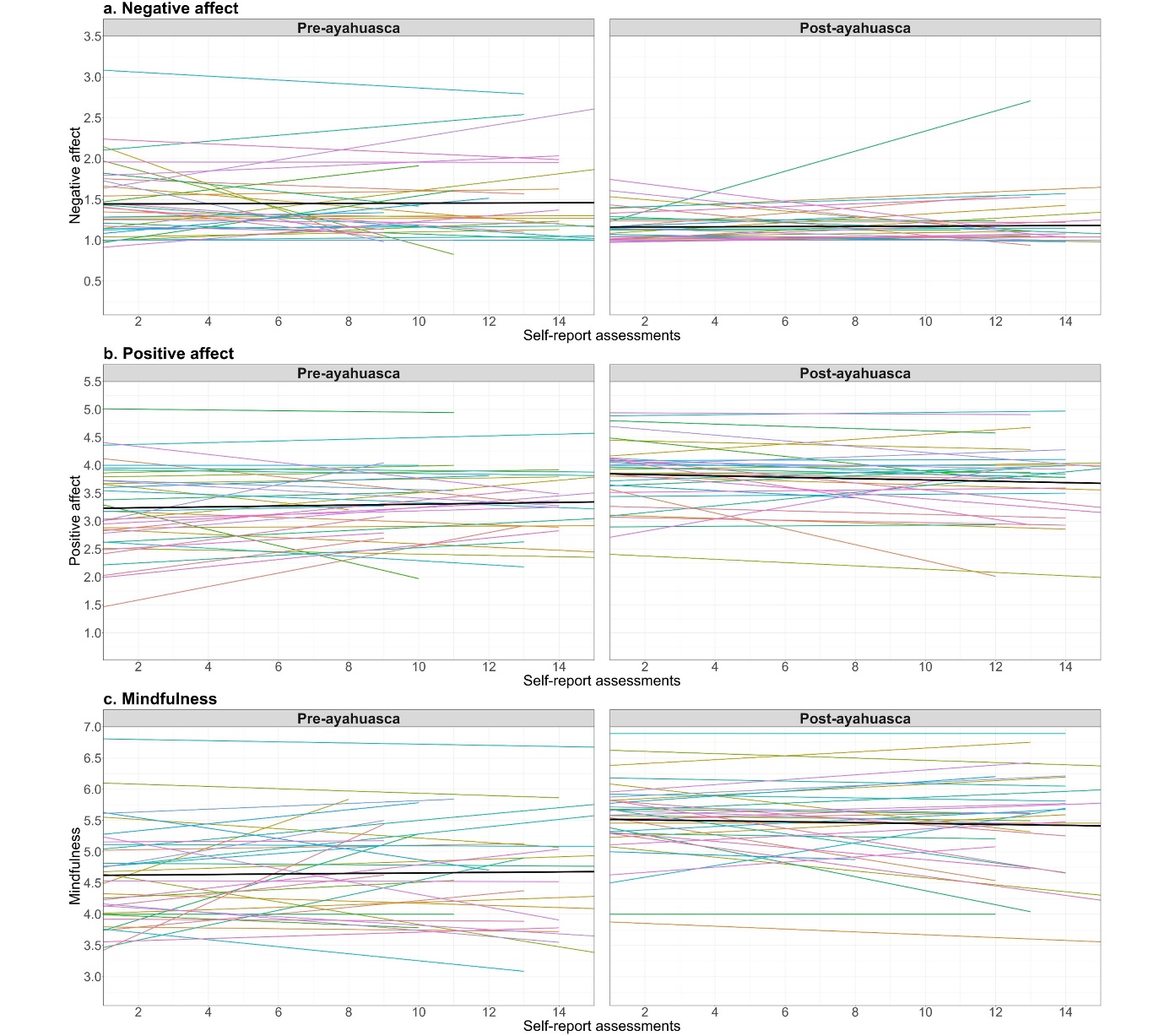


**Fig S1** Spaghetti plots of individual and mean linear trajectories and regression lines of subacute effects, pre- and post-ayahuasca retreat.
